# Neonatal Antibiotic Treatment Is Associated With an Altered Circulating Immune Marker Profile at 1 Year of Age

**DOI:** 10.3389/fimmu.2019.02939

**Published:** 2020-01-10

**Authors:** Berthe C. Oosterloo, Belinda van't Land, Wilco de Jager, Nicole B. Rutten, Margot Klöpping, Johan Garssen, Arine M. Vlieger, Ruurd M. van Elburg

**Affiliations:** ^1^Department of Pediatrics, Emma Children's Hospital, Amsterdam UMC, University of Amsterdam, Amsterdam, Netherlands; ^2^Center for Translational Immunology, University Medical Centre Utrecht, Utrecht, Netherlands; ^3^Danone Nutricia Research, Utrecht, Netherlands; ^4^St. Antonius Hospital, Department of Pediatrics, Nieuwegein, Netherlands; ^5^Utrecht Institute for Pharmaceutical Sciences (UIPS), Utrecht University, Utrecht, Netherlands

**Keywords:** biomarkers, immune development, infant, antibiotics, infantile colic, eczema

## Abstract

**Background:** Neonatal antibiotics disturb the developing gut microbiome and are therefore thought to influence the developing immune system, but exact mechanisms and health consequences in later life still need to be elucidated. Therefore, we investigated whether neonatal antibiotics influence inflammatory markers at 1 year of age. In addition, we determined whether health problems during the first year of life, e.g., allergic disorders (eczema and wheezing) or infantile colics, were associated with changes in the circulating immune marker profile at 1 year of age.

**Methods:** In a subgroup (*N* = 149) of the INCA-study, a prospective birth-cohort study, a blood sample was drawn from term born infants at 1 year of age and analyzed for 84 immune related markers using Luminex. Associations of antibiotic treatment, eczema, wheezing, and infantile colics with immune marker concentrations were investigated using a linear regression model. The trial is registered as NCT02536560.

**Results:** The use of broad-spectrum antibiotics in the first week of life, was significantly associated with different levels of inflammatory markers including sVCAM-1, sCD14, sCD19, sCD27, IL-1RII, sVEGF-R1, and HSP70 at 1 year of age. Eczema was associated with decreased concentrations of IFNα, IFNγ, TSLP, CXCL9, and CXCL13, but increased concentrations of CCL18 and Galectin-3. Wheezing, independent of antibiotic treatment, was positively associated to TNF-R2 and resistin. Infantile colics were positively associated to IL-31, LIGHT, YKL-40, CXCL13, sPD1, IL1RI, sIL-7Ra, Gal-1, Gal-9, and S100A8 at 1 year of age, independent of early life antibiotic treatment.

**Conclusion:** In this explorative study, we identified that neonatal antibiotics are associated with immunological alterations at 1 year of age and that, independent of the antibiotic treatment, infantile colics were associated with alterations within gut associated markers. These findings support the importance of the first host microbe interaction in early life immune development.

## Introduction

Early immune development is influenced by many different pre- and post-natal factors (1). Delivery mode, infant feeding, exposure to the environment, and antibiotic treatment are all early life exposures known to influence the developing immune system (2–6). One of the mechanisms by which these early life factors can influence the immune system is probably through their impact on the developing gut microbiome (1). The microbiome development starts right after birth, and is very dynamic during the early life period (7). When the microbiome development is disrupted, this may impact immune development, with long term health effects such as development of asthma and/or allergies but also of inflammatory bowel disease (IBD), type 1 diabetes (6). One of the most important and well-known factors that disturbs the normal microbiome development is antibiotic treatment in early life (5, 6). Antibiotics are nevertheless the most frequently prescribed drug for neonates (8).

The INCA study, a prospective birth-cohort study, was designed to investigate the long-term clinical, immunological and microbial effects of antibiotic treatment in the first week of life. The primary hypothesis was that children treated with antibiotics in the first week of life would have eczema more often (9). In this cohort, we previously demonstrated an increased risk for infantile colic and wheezing in the first year of life in children treated with antibiotics during the first week of life, but not for eczema (10). Although epidemiologic studies show a direct association between the use of antibiotics during the first year of life and the risk for development of asthma and other diseases later in life (6, 11), the analysis of inflammatory biomarkers in these otherwise healthy infants remains rather limited.

We hypothesized that antibiotic treatment in the first week of life may induce certain levels of immunological misbalance, resulting in alteration of circulating immune marker profile at 1 year of age. Aim of this explorative study was to measure the circulating immune marker profile at 1 year of age in a subgroup of the INCA study, with or without antibiotic treatment in the first week of life. In addition, we explored whether health problems such as allergic disorders (eczema or wheezing) or infantile colics in the first year of life were associated with changes in this circulating immune marker profile in children at 1 year of age.

## Methods

### Study Design

The INCA (INtestinal microbiota Composition after Antibiotic treatment in early life) study is a prospective birth-cohort study. Design, inclusion and exclusion criteria of this cohort have been published previously (9). Between August 2012 and January 2015, term-born infants (≥36 weeks of gestation) were recruited from the maternity and neonatal wards of four teaching hospitals in the Netherlands. Antibiotic treatment was started at the pediatrician's discretion, according to hospital protocol for suspected early onset neonatal infection and based on the Dutch guideline for early onset sepsis (12). In general, infants with suspicion of infection received broad-spectrum antibiotics (a combination of gentamycin and a penicillin-derivative), after a blood culture was taken. In case of a negative blood culture, combined with a low clinical suspicion of infection and low c-reactive protein, antibiotics were discontinued after 2–3 days, otherwise antibiotics were continued for 7 days. All term born infants staying in the hospital for at least 24 h were eligible for inclusion. Exclusion criteria were severe congenital malformations, severe infection needing transfer to a neonatal intensive care unit, and insufficient knowledge of the Dutch language. Around the age of 1 year, children visited the outpatient clinic for follow-up. During this visit, a blood sample was obtained if the parents had given additional informed consent. After centrifugation, serum samples were aliquoted and stored at −80°C until further use. Informed consent was obtained from both parents at inclusion. The study was approved by the ethical board of the St. Antonius Hospital in Nieuwegein. The study was registered as NCT02536560.

### Data Collection

Atopic diagnoses were recorded as published previously (10). In short, parents kept a diary and reported symptoms of atopic disorders and crying for more than 3 h per day. Doctor diagnosed eczema (DDE) in this study was defined as eczema confirmed by the general practitioner, investigated via the General Practitioner electronic medical database using the International Classification of Primary Care (ICPC) (13). An episode of wheezing was defined as wheezing present for at least two consecutive days. Infantile colics were defined according to the Rome III criteria with parent-reported crying for ≥3 h of crying per day, ≥3 days within a week, within the first 3 months of life (14).

### Cytokine Measurements

Measurements of immune-related markers (*n* = 84) (Supplementary Table 1) were performed using an in-house developed and validated multiplex immunoassay based on Luminex technology (xMAP, Luminex Austin, TX, USA). The assay was performed as described by Scholman et al. (15). In short, a-specific heterophilic immunoglobulins were pre-absorbed from all samples with heteroblock (Omega Biologicals, Bozeman MT, USA). Next, samples were incubated with antibody-conjugated MagPlex microspheres for 1 h at room temperature with continuous shaking, followed by 1 h incubation with biotinylated antibodies, and 10 min incubation with phycoerythrin-conjugated streptavidin diluted in high performance ELISA buffer (HPE, Sanquin, the Netherlands). Acquisition was performed with the Biorad FlexMAP3D (Biorad laboratories, Hercules, USA) in combination with xPONENT software version 4.2 (Luminex). Data was analyzed by 5-parametric curve fitting using Bio-Plex Manager software, version 6.1.1 (Biorad). Potential cross-reactive samples were identified using a negative control (15) and were excluded from analysis. After determining the cytokine/chemokine serum levels, the out of range (OOR) data have been imputed with the lower limit of quantification (LLOQ) in lower OOR threshold or the upper limit of quantification (ULOQ) in the upper OOR threshold by using assay characteristics (LLOQ and ULOQ) as previously published (15). If ≥40% of the data was imputed for the same biomarker, and equally divided over the compared outcome, the biomarker was excluded from further analysis.

### Statistical Analyses

Basic descriptive statistics (Mann Whitney U- or X-squared tests) were used to describe the patient population. As described previously, an unsupervised hierarchal clustering analysis, with min-max normalization per protein, was performed to investigate the discriminative potential of a single or a combination of proteins (16).

Not-normally distributed cytokines and chemokines were log-transformed to achieve a Gaussian distribution. With a linear regression the association between (log-transformed) cytokines and chemokines and an antibiotic course in the first week of life was investigated. Next, we investigated the association of the cytokines and chemokines and doctor's diagnosed eczema, wheezing and infantile colic. Wheezing and infantile colic analyses were additionally adjusted for antibiotic treatment in the first week of life as this was shown before to be associated. Doctor's diagnosed eczema was not associated to the antibiotic course in the first week of life and therefore these analyses were not adjusted for antibiotic treatment in the first week of life. Back -transformed βs are shown for the log-transformed variables.

As we consider this study an exploratory, hypothesis-generating study, *p* < 0.05 were considered significant. We do acknowledge, however, the problem of multiple testing in this study, therefore we focus mainly on the associations with a *p* < 0.01.

Statistical analyses were performed using either IBM SPSS Statistics 24, R statistics version 3.5.1, Omniviz 6.1.2, or Graphpad Prism 7.

## Results

### Baseline Characteristics

Baseline characteristics were comparable between the complete INCA-cohort (*n* = 436) and the subpopulation analyzed in this study of which a sufficient serum sample was obtained (*n* = 167, Table 1). Of these 167 samples, 18 were excluded from further analysis due to cross reactivity, leaving 149 samples from 149 infants suitable for analysis (Luminex-group). Of all markers, 14 were excluded as they were ≥40% below the LLOQ (Supplementary Table 1). No significant differences were found in describing characteristics of the children with and without antibiotics (Table 1).

**Table 1:** Participant characteristics of samples analyzed with luminex compared to the whole cohort.

		**Luminex cohort (*N* = 149)**	**AB– (*N* = 95)**	**AB+ (*N* = 54)**	**INCA clinical cohort (*N* = 436)**
Gender	Male (%)	90 (60.4%)	56 (58.9)	34 (63.0)	237 (54.4%)
Gestational age	(SD)	39.7 (1.5)	39.4 (1.5)	40.0 (1.2)	39.7 (1.5)
Birthweight	(SD)	3,552 (544)	3,465 (557)	3,707 (489)	3,526 (546)
Delivery mode	Vaginal (%)	111 (74.5%)	68 (71.6)	43 (79.6)	599 (68.6%)
Breastfed exclusive	0 months (%)	33 (22.1%)	42 (44.2)	31 (57.4)	99 (22.7%)
	1–3 months	63 (42.3%)	26 (27.4)	7 (13.0)	181 (41.5%)
	>3 months	53 (35.6%)	27 (28.4)	16 (29.7)	156 (35.8%)
Antibiotics (< day 7)	(%)	54 (36.2%)			151 (34.6%)
Infantile colic	(%)	26 (17.4%)	14 (14.7)	12 (22.2)	74 (17.0%)
Eczema	(%)	20 (13.4%)	15 (15.8)	5 (9.3)	58 (13.3%)

### Neonatal Antibiotic Treatment and Circulating Immune Profile at 1 Year of Age

Antibiotic treatment in the first week of life was significantly associated with higher or lower concentrations of IL-12 (β 0.63, 95% CI 0.45, 0.89), CCL2 (β −10.14, 95%CI −19.35, −0.92), CXCL4 (β 0.80, 95% CI 0.67, 0.94), sVCAM-1 (β −0.66, 95% CI −1.27, −0.06), sCD14 (β 1.09, 95% CI 1.02, 1.16), sCD19 (β 0.20, 95% CI 0.09, 0.46), sCD27 (β 0.44, 95% CI 0.28, 0.69), TNF-R1 (β 0.62, 95% CI 0.42, 0.93), sVEGF-R1 (β 0.74, 95% CI 0.58, 0.94), E-selectin (β −11.52, 95% CI −23.01, −0.03), and HSP70 (β 0.36, 95% CI 0.21, 0.62) (Table 2).

**Table 2:** The significantly altered immune marker concentrations in one of the associations.

**Immune marker**		**AB+** **vs AB–**				**Doctors diagnosed eczema**				**Wheezing (adjusted for AB)**				**Infantile colics (adjusted for AB)**			
			**95%CI**				**95%CI**				**95%CI**				**95%CI**		
**Unit**	**Marker**	**β**	**Low**	**Up**	***p*-value**	**β**	**Low**	**Up**	***p*-value**	**β**	**Low**	**Up**	***p*-value**	**β**	**Low**	**Up**	***p*-value**
pg/ml	IL-12	0.63	0.45	0.89	0.010	1.09	0.66	1.81	n.s.	1.27	0.89	1.80	n.s.	1.34	0.86	2.09	n.s.
pg/ml	IL-17	0.83	0.67	1.03	n.s.	0.83	0.61	1.13	0.01	0.81	0.65	1.01	n.s.	1.04	0.79	1.38	n.s.
pg/ml	IL-17F	0.87	0.68	1.10	n.s.	0.87	0.62	1.22	n.s.	1.30	1.02	1.65	n.s.	1.52	1.13	2.04	0.006
pg/ml	IL-22	0.73	0.50	1.07	n.s.	1.43	0.83	2.46	n.s.	1.04	0.71	1.53	n.s.	1.62	1.00	2.62	0.050
pg/ml	IL-31	0.92	0.65	1.30	n.s.	0.58	0.36	0.94	0.027	1.03	0.72	1.45	n.s.	1.72	1.11	2.65	0.015
pg/ml	IL-33	0.71	0.54	0.92	n.s.	0.92	0.62	1.36	n.s.	1.05	0.80	1.38	n.s.	1.61	1.15	2.25	0.006
pg/ml	IFNa	0.97	0.61	1.55	n.s.	0.44	0.23	0.83	0.012	0.81	0.51	1.29	n.s.	1.26	0.70	2.27	n.s.
pg/ml	IFNg	1.30	0.80	2.13	n.s.	0.50	0.25	0.99	0.048	0.76	0.47	1.25	n.s.	1.37	0.73	2.57	n.s.
pg/ml	TSLP	1.19	0.75	1.90	n.s.	0.47	0.25	0.91	0.025	0.74	0.46	1.18	n.s.	1.44	0.80	2.62	n.s.
pg/ml	LIGHT	0.94	0.64	1.37	n.s.	0.91	0.54	1.56	n.s.	1.13	0.77	1.65	n.s.	1.70	1.06	2.74	0.028
ng/ml	YKL-40	0.96	0.81	1.14	n.s.	0.97	0.76	1.24	n.s.	1.06	0.89	1.26	n.s.	1.25	1.01	1.55	0.044
pg/ml	^*^CCL2	−10.14	−19.35	−0.92	0.031	−5.24	−18.42	7.93	n.s.	7.70	−1.51	16.91	n.s.	6.92	−4.79	18.63	n.s.
ng/ml	CCL18	1.03	0.88	1.21	n.s.	1.26	1.01	1.56	0.040	1.08	0.92	1.27	n.s.	1.03	0.85	1.26	n.s.
μg/ml	CXCL4	0.80	0.67	0.94	0.009	0.98	0.77	1.25	n.s.	0.94	0.80	1.12	n.s.	0.97	0.79	1.21	n.s.
pg/ml	CXCL9	1.11	0.86	1.42	n.s.	0.64	0.45	0.91	0.014	1.03	0.80	1.33	n.s.	1.02	0.74	1.40	n.s.
pg/ml	CXCL13	1.09	0.91	1.30	n.s.	0.76	0.59	0.98	0.034	1.09	0.91	1.31	n.s.	1.28	1.02	1.61	0.034
pg/ml	^*^sPD1	−81.83	−197.41	33.75	n.s.	−70.59	−234.25	93.06	n.s.	61.96	−54.15	178.08	n.s.	185.17	40.75	329.59	0.012
ng/ml	sCD19	0.20	0.09	0.46	0.000	0.86	0.25	2.91	n.s.	1.33	0.58	3.06	n.s.	1.34	0.47	3.87	n.s.
pg/ml	sCD27	0.44	0.28	0.69	0.000	0.61	0.31	1.17	n.s.	1.01	0.64	1.60	n.s.	1.44	0.81	2.56	n.s.
pg/ml	TNF-R1	0.62	0.42	0.93	0.020	0.67	0.38	1.18	n.s.	1.54	1.04	2.28	0.033	1.31	0.79	2.17	n.s.
ng/ml	^*^TNF-R2	−0.13	−0.30	0.05	n.s.	−0.11	−0.35	0.14	n.s.	0.14	−0.04	0.31	n.s.	0.23	0.01	0.45	0.037
ng/ml	sIL-7Ra	0.76	0.52	1.11	n.s.	1.03	0.60	1.77	n.s.	1.04	0.71	1.52	n.s.	1.74	1.08	2.80	0.023
ng/ml	sVEGF-R1	0.74	0.58	0.94	0.014	0.94	0.66	1.32	n.s.	1.18	0.93	1.50	n.s.	1.21	0.89	1.64	n.s.
ng/ml	^*^Gal-1	−1.57	−3.53	0.39	n.s.	−0.31	−3.10	2.48	n.s.	1.07	−0.90	3.04	n.s.	3.92	1.50	6.34	0.002
ng/ml	Gal-3	0.86	0.71	1.04	n.s.	1.34	1.02	1.75	0.038	1.06	0.87	1.29	n.s.	1.19	0.93	1.52	n.s.
ng/ml	Gal-9	0.91	0.76	1.09	n.s.	0.96	0.75	1.24	n.s.	1.19	1.00	1.42	n.s.	1.31	1.05	1.63	0.018
ng/ml	^*^E-selectin	−11.52	−23.01	−0.03	0.049	5.98	−10.40	22.37	n.s.	5.56	−5.99	17.11	n.s.	6.38	−8.24	21.01	n.s.
ng/ml	S100A8	0.96	0.71	1.30	n.s.	1.06	0.69	1.61	n.s.	1.16	0.86	1.57	n.s.	1.51	1.03	2.20	0.034
ng/ml	HSP70	0.36	0.21	0.62	0.000	0.95	0.43	2.11	n.s.	1.25	0.73	2.16	n.s.	1.38	0.69	2.75	n.s.
ng/ml	Resistin	1.00	0.83	1.19	n.s.	0.99	0.77	1.28	n.s.	1.20	1.00	1.43	0.048	0.65	−1.16	2.46	n.s.

*n.s. is non-significant*.

* *Reflects the values that were not log-transformed*.

**Figure 1:**
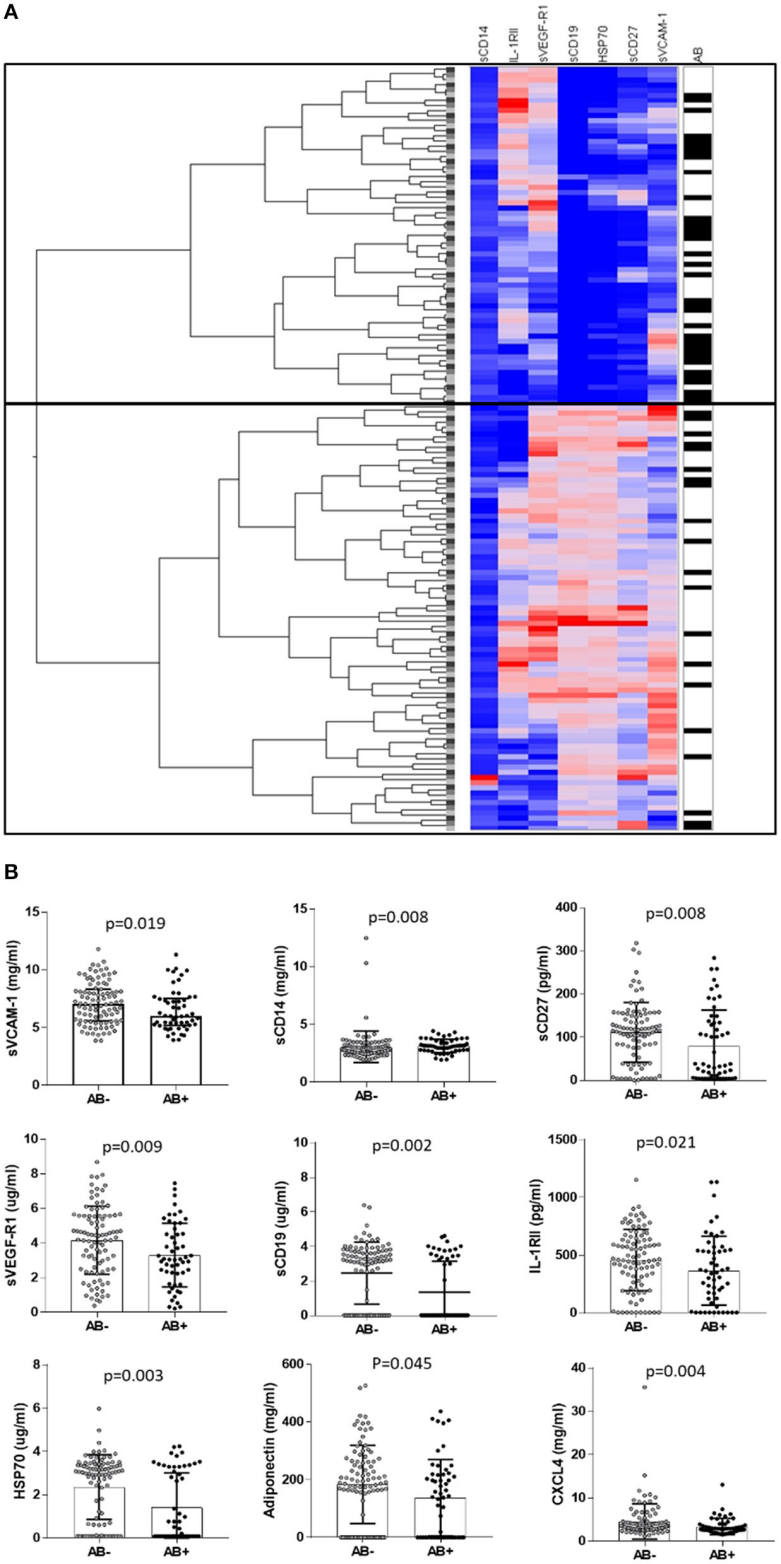
**(A)** Hierarchical differentiating clustering, differentiating factor is Antibiotic treatment in first week of life; markers associated are sVCAM-1, sCD14, sCD19, sCD27, IL-1RII, sVEGF-R1, and HSP70. **(B)** Distribution of immune markers appearing in the hierarchical cluster between the children treated with antibiotics in the first week of life compared to those who were not.

Moreover, the concentrations of sVCAM-1, sCD14, sCD19, sCD27, IL-1RII, sVEGF-R1, and HSP70 were significantly (*p* < 0.05) associated with neonatal antibiotic treatment in a differentiating cluster (Figures 1A,B). Overall, some of inflammatory markers measured showed significant differences between the AB+ and AB– groups (Table 3).

**Table 3:** Absolute values of the markers significantly differing in children treated without (AB–) and with (AB+) antibiotics in the first week of life, given in median [inter quartile range (IQR)].

		**AB–** ***n*** **=** **95**			**AB+** ***n*** **=** **54**			
**/ml**		**Median**	**IQR**		**Median**	**IQR**		***p*-value**
pg	IL-22	3.7	3.7	7.7	3.7	1.5	4.0	<0.05
pg	CCL2	55	38	81	44	31	71	<0.05
μg	CXCL4	3.5	2.8	4.5	2.9	2.3	3.9	<0.01
μg	sVCAM-1	7.1	5.6	8.3	6.0	5.2	7.5	<0.05
μg	sCD14	2.8	2.5	3.2	3.1	2.8	3.6	<0.01
ng	sCD19	3.2	0.0	3.7	0.0	0.0	3.5	<0.01
pg	sCD27	114	75	154	36	5.4	135	<0.01
pg	IL-1RII	461	266	649	346	115	543	<0.05
pg	TNF-R1	99	47	196	83	30	134	<0.05
ng	sVEGF-R1	4.4	2.5	5.6	3.1	2.1	4.6	<0.01
ng	HSP70	3.0	0.6	3.4	0.5	0.1	3.3	<0.01
μg	Adiponectin	190	0.2	265	142	0.2	219	<0.05

### Health Problems in the First Year and Circulating Immune Profile at 1 Year

The incidence of DDE in the infants in the subgroup was comparable to the incidence of DDE in the total INCA clinical cohort (13.4 vs. 13.3%, respectively, Table 1). DDE was significantly associated with lower concentrations of IFNα (β 0.44, 95% CI 0.23, 0.83), IFNγ (β 0.50, 95% CI 0.25, 0.99), TSLP (β 0.47, 95% CI 0.25, 0.91), CXCL9 (β 0.64, 95% CI 0.45, 0.91), and CXCL13 (β 0.76, 95% CI 0.59, 0.98). In children with DDE there was a positive association with CCL18 (β 1.26, 95% CI 1.01, 1.56) and Galectin-3 (β 1.34, 95% CI 1.02, 1.75) (Table 2).

Incidence of wheezing in the subgroup was comparable to the incidence of wheezing in the total INCA clinical cohort (36.2 vs. 34.2%). In children that wheezed both TNF-R2 (β 1.54, 95% CI 1.04, 2.28) and resistin (β 1.20, 95% CI 1.00, 1.43) were positively associated (Table 2).

Incidence of infantile colics within the subgroup was comparable to the total INCA clinical cohort (17.0 vs. 17.4%, respectively). Interestingly, the positively significantly associated markers are known as inflammatory and gut associated immune markers (Table 2); Il-22 (β 1.62, 95% CI 1.00, 2.62), LIGHT (β 1.70, 95% CI 1.06, 2.74), YKL-40 (β 1.25, 95% CI 1.01, 1.55), CXCL13 (β 1.28, 95% CI 1.02, 1.61), sPD1 (β 185.17, 95% CI 40.75, 329.59), sIL-7ra (β 1.74, 95% CI 1.08, 2.80), Gal-1 (β 3.92, 95% CI 1.50, 6.34), Gal-9 (β 1.31, 95% CI 1.05, 1.63), S100A8 (β 1.51, 95% CI 1.03, 2.20). The only negatively associated marker was TNF-R2 (β 0.23, 95% CI 0.01, 0.45).

## Discussion

In this INCA-cohort, we found that children treated with neonatal antibiotics (in the first week of life) have a different circulating immune marker profile at 1 year of age compared to children not exposed to neonatal antibiotics. In addition, children who suffered from infantile colics during the first 3 months of their life, had increased (gut-associated) inflammatory markers (like IL-33 and S100A8 and Galectin 1) at 1 year of age. Moreover, we found that children with doctor's diagnosed eczema had limited capacity to induce Th1 cytokines (like IFN-gamma and CXCL9) and more eczema/skin related marker CCL18 (PARC). To our knowledge, this is the first study that explored the impact of antibiotic treatment in the first week of life in term-born infants on the circulating immune marker profile at 1 year of age.

Diversity of the early developing gut microbiota and repeated exposure to new bacterial antigens seems to be more important for normal immune maturation than the distribution of specific species (17). Aberrant immune maturation in early infancy has been linked to limited intensity and reduced diversity of microbial stimulation (18). Consequently, antibiotic treatment can be seen as a risk factor for development of altered microbial diversity in early life, with the potential of altered immune development (5). Neonatal nutritional status is one of the important environmental factors in this process of immune maturation. However, no association between the duration of breastfeeding and clinical characteristics or immune markers could be detected between AB+ and AB– study groups. Due to the explorative nature of the study, it is difficult to determine the role of changes in individual cytokines. However, some interesting markers could be related to an altered microbial management capacity of the immune system in infants receiving antibiotics. For instance, changes in the levels of both sCD14 and CXCL4 concentrations suggest higher levels of microbial components within circulation (19, 20). Moreover, whereas CD19 and CD27 are linked to B-cell development, known to be involved in the dynamic immune maturation period in children, the concentrations of sCD19 and sCD27 were lower in infants receiving antibiotics compared to healthy controls. This might be a reflection of an altered B-cell development, suggesting a link between the sIgA development and early life microbiome establishment (21). Subsequently, the presence of reduced levels of individual circulating inflammation related receptors (like IL1-RII, TNF-R1, and sVEGF-R1) are indicative for an altered immune development in infants receiving early life antibiotics. Clustering of these markers showed a different immune development at 1 year of age in infants who received antibiotic treatment in the first week of life. This in turn is illustrative for the importance and understanding of the long-lasting effects of several environmental factors which occur early in life and are known to be associated with changes later in life.

One of the features of an altered immune development or immune dysbiosis in early life is the development of allergic disorders. Although, within the INCA study, no increased risk was shown for doctor's diagnosed eczema after neonatal antibiotic treatment at 1 year of age (10), increased concentrations of Gal-3, CCL17 (TARC), and CCL18 (PARC) were detected within the infants with DDE. These markers are associated with either skin inflammation, Gal-3 (22), or eczema severity, TARC, and PARC (23). It is interesting to note that characteristic eczema severity markers like TARC and PARC, were not associated with the parental reported eczema (data not shown). Parental reported eczema (PRE) is considered to be less specific for the (real) presence of eczema. Increased concentrations of Gal-3 in children with PRE may reflect another form of skin inflammation. Non-specific eczema-like symptoms can be caused by a wide range of factors, all associated with their own immune marker profile.

One additional atopy-related outcome evaluated within the INCA study was wheezing. Infants treated with neonatal antibiotics had an increased risk for wheezing in the first year of life (10). Wheezing is, however, a non-specific outcome, as many young children suffer from an episode of wheezing, often due to viral infections. Yet, it is used as a predictor for the development of asthma. Its non-specific nature (i.e., can both represent a respiratory infection as well as an allergic response), might be an explanation for the absence of clear differences in circulating immune marker profile between children who suffered from wheezing and those who did not.

We previously showed that neonatal antibiotic treatment is a contributing risk factor for development of infantile colics in the first year of life (10). The etiology of infantile colics is largely unknown, but may be related to gut function immaturity, low grade inflammation, gut dysmotility, food allergy and, similarly to parental stress, anxiety, and exhaustion (24–26). Moreover, gut microbiota alterations, associated with intestinal barrier dysfunction, are reported in colicky infants (27). The effect of neonatal antibiotics on the circulating immune marker profile in children with infantile colics was not investigated due to the small size of the subgroup. However, we additionally accounted for neonatal antibiotic treatment in the analyses. Because infantile colics usually resolved without specific treatment around 3 months of age, it is very interesting that at 1 year of age gut associated inflammatory markers such as IL-31, Gal-1, Gal-9, S100A8 were increased in children that previously had infantile colics compared to those who had not. These markers are reported to be both gut associated and allergy associated (28–31). Given this finding, it is interesting that recent studies have focused on the association between infantile colics and gastrointestinal disorders later in life, suggesting that infantile colics may have long term consequences (32, 33).

Very limited information is available regarding the levels of inflammatory markers within healthy infants. However, some of the markers are associated with increased inflammation status, specifically acting as marker for disease activity. Regarding the marker profile associated with infantile antibiotics use, only associations can be made. i.e., it is known that after exposure to bacterial endotoxin, monocytes release sCD14 and plasma levels are altered in conditions associated with microbial translocation such as insulin resistance, liver inflammation, and cardiovascular disease (34–36). A marker such as Heat Shock Protein 70 (HSP) is a stress-responsive protein (37). In addition, sCD27 may act to differentiate activated memory or recently antigen-experienced B cells (38). In addition, eczema was associated with decreased concentrations of IFNα, IFNγ, TSLP, CXCL9, and CXCL13, but with increased concentrations of CCL18 and Galectin-3. Most of the potential biomarkers described thus far in the literature for the development of eczema related disorders, like atopic dermatitis, are more or less related to the issue of severity and their changes during the therapeutic regimen. Among these, thymus and activation-regulated chemokine (CCL17), macrophage derived chemokine (CCL22), cutaneous T-cell-attracting chemokine (CCL27), IL-31, IL-33, IL-22, LL37, IL-18, IL-16, pulmonary and activation-regulated chemokine (CCL18), periostin, and the soluble IL-2 receptor are describing different phenotypes of disease (39). Although the very initial stage of infantile atopic dermatitis is very difficult to diagnose, the typical eczematous lesions on respective localizations emerge around the second month of life and may or may not develop into an allergic phenotype (40). Limited information is available regarding the levels of these individual markers within infants and their link to eczema. Moreover, regarding Infantile colics which were positively associated to IL-31, LIGHT, YKL-40, CXCL13, sPD1, IL1RI, sIL-7Ra, Gal-1, Gal-9, and S100A8 at 1 year of age, the impairment of the intestinal mucosal immunity significantly increases the risk of acute and chronic diseases. Within gastrointestinal diseases, a galectin-specific signature in the gut delineates Crohn's disease and ulcerative colitis from other human inflammatory intestinal disorders (41). Moreover, IL-31 is a Th2 type cytokine, which may be involved in the immune and inflammatory responses of the intestinal mucosa. During inflammation, S100A8/A9 is released actively and exerts a critical role in modulating the inflammatory response by stimulating leukocyte recruitment and inducing cytokine secretion (42). Lymphoid chemokines, such as CCL19, CCL21, and CXCL13, have an important role in the formation of secondary lymphoid organs and Peyers Patches (43, 44) These associations collectively warrant the notification within our study that the inflammatory cytokine profile has been altered, which is indicative for possible change in immune balance development.

A limitation of this study is the cross-sectional design. Ideally, more sampling time points would have given us the possibility to analyze time-dependent changes in immune marker development. Additionally, due to the sample size and cross-sectional design, no additional confounder adjustments were made, such as for the mode of delivery, which is known to contribute to an altered gut microbiome composition in the first weeks and months after birth (45). We are aware that many factors could influence the development of the immune marker profile during the first year of life other than “only” neonatal antibiotic treatment, e.g., other antibiotic courses in the first year of life. Nevertheless, this study does provide novel insights regarding the immune marker profile of a relatively healthy 1 year old population. While this is one of the largest groups of young children described in literature so far, the design of the study and sample size do not allow for determination of causal associations. However, it may help in elucidating the consequences of neonatal antibiotic treatment for the risk of developing infantile colics and atopic disorders. Although our biomarker study does show alterations within inflammatory biomarkers associated with the early life use of antibiotics, it has not been designed for development of predictive biomarker profiles. The impact of antibiotic use on clinical features has been demonstrated, which now seems reflected in small but significant alteration of biomarker profiles. However, due to the fact that a diversity of markers are changing, as not a single one stands out, combined with the small sample size, we have to conclude that our understanding of the impact of early life antibiotics use remains limited. Aforementioned factors refrain us from developing a predictive biomarker set in this regard.

In conclusion, this explorative study shows that the circulating immune marker profile at 1 year of age is affected by antibiotic treatment during the first week of life. Furthermore, infants who suffered from infantile colics in the first 3 months of life show increased gut-associated immune markers at 1 year of age. Follow-up of this cohort can elucidate whether these increased gut associated markers predispose them for gastro-intestinal disorders later in life. The results of this explorative study imply the existence of long-term negative consequences of antibiotic treatment on immune development, possibly resulting in negative health effects later in life.

## Data Availability Statement

The dataset generated to support the findings of this study are available from the corresponding author, upon reasonable request.

## Ethics Statement

The studies involving human participants were reviewed and approved by the ethical board of the St. Antonius Hospital in Nieuwegein. Written informed consent to participate in this study was provided by the participants' legal guardian/next of kin.

## Author Contributions

BO, BL, and RE: data analysis and writing the manuscript. WJ and MK: sample analysis and critical reading of the manuscript. NR and AV: conception of the study, data collection, and critical reading of the manuscript. JG: critical reading the manuscript.

### Conflict of Interest

JG is head of the Division of Pharmacology, Utrecht Institute for Pharmaceutical Sciences, Faculty of Science at the Utrecht University, and partly employed by Nutricia Research. BL is employed by Nutricia Research and as indicated by the affiliations, leading a strategic alliance between Nutricia Research and University Medical Center Utrecht/Wilhelmina Children's Hospital. The remaining authors declare that the research was conducted in the absence of any commercial or financial relationships that could be construed as a potential conflict of interest. The handling editor declared a past co-authorship with one of the authors RE.
